# Global review of shorebird tracking data to identify research gaps and conservation priorities

**DOI:** 10.1111/cobi.70211

**Published:** 2026-01-14

**Authors:** Grégoire Michel, Josh Nightingale, Martin Beal, Alice Bernard, Maria P. Dias, José A. Alves

**Affiliations:** ^1^ Centre for Ecology, Evolution and Environmental Changes (CE3C) & CHANGE – Global Change and Sustainability Institute, Department of Animal Biology, Faculdade de Ciências Universidade de Lisboa, Campo Grande Lisboa Portugal; ^2^ South Iceland Research Centre University of Iceland Laugarvatn Iceland; ^3^ Department of Biology & CESAM—Centre for Environmental and Marine Studies University of Aveiro, Campus de Santiago Aveiro Portugal; ^4^ CEFE, Univ Montpellier, CNRS, EPHE, IRD Montpellier France

**Keywords:** animal migration, biodiversity conservation, biologging, biotelemetry, movement ecology, open data, waders, biotelemetría, bioregistro, conservación de la biodiversidad, datos abiertos, ecología del movimiento, migración animal, vadeadores

## Abstract

Tracking has enabled rapid advances in knowledge of the movement behavior and habitat use of shorebirds and is thus making a growing contribution to their conservation. However, realizing the full potential that tracking holds for conservation involves understanding what has been performed on shorebirds to date and identifying regional and taxonomic knowledge gaps. To this end, we reviewed the literature on 195 species across 10 shorebird families. We determined the number of shorebird tracking studies published over time, types of tracking devices used, reporting rates for data archiving in online repositories, and coverage of the major flyways by the data collected. Using Movebank, we further identified tracked species that have not appeared in the literature. We included 351 peer‐reviewed publications in the review. Tracking data were lacking for 50% of the species reviewed. Considerably more tracking studies were conducted in temperate regions and in flyways that include wealthy countries than in the tropics. Of the 351 publications, 26.9% reported data were archived in an online repository, although the annual rate increased over time. We identified 16 species whose conservation needs and a lack of data make them relevant priorities for future tracking. Improving data archiving practices and coordination around tag deployment to cover understudied regions is key to maximizing the utility of tracking for shorebird research and conservation.

## Abstract (es)

Revisión mundial de los datos de rastreo de aves costeras para identificar los vacíos de investigación y las prioridades de conservación

**Resumen:**

El rastreo ha permitido avances rápidos en el conocimiento del comportamiento migratorio y el uso del hábitat de las aves costeras, lo que contribuye cada vez más a su conservación. Sin embargo, para aprovechar todo el potencial que el rastreo tiene para la conservación, es necesario comprender lo que se ha hecho hasta la fecha con las aves costeras e identificar los vacíos de conocimiento regionales y taxonómicos. Con este fin, revisamos la bibliografía sobre 195 especies de 10 familias de aves costeras. Determinamos el número de estudios de rastreo de aves costeras publicados a lo largo del tiempo, los tipos de dispositivos de rastreo utilizados, las tasas de notificación para el archivo de datos en repositorios en línea y la cobertura de las principales rutas migratorias por los datos recopilados. Utilizamos Movebank para identificar las especies rastreadas que no han aparecido en la bibliografía. Incluimos 351 publicaciones revisadas por pares en la revisión. El 50% de las especies revisadas carecía de datos de seguimiento. Una mayoría considerable de estudios de rastreo se realizó en regiones templadas y en rutas migratorias que incluyen países ricos, más que en los trópicos. De las 351 publicaciones, el 26.9% informó que los datos se archivaron en un repositorio en línea, aunque la tasa anual aumentó con el tiempo. Identificamos 16 especies cuyas necesidades de conservación y la falta de datos las convierten en prioridades relevantes para el seguimiento a futuro. Mejorar las prácticas de archivo de datos y la coordinación en torno al despliegue de etiquetas para cubrir las regiones poco estudiadas es clave para maximizar la utilidad del seguimiento para la investigación y la conservación de las aves playeras.

## INTRODUCTION

Since the late 1950s, the movements of wild animals have been tracked using miniature electronic devices, and there has been rapid technological development and proliferation of such devices in recent decades (Kays et al., 2015; Ropert‐Coudert & Wilson, 2005). Tracking technologies (e.g., global positioning systems [GPSs] and platform transmitter terminals [PTTs]) are providing spatiotemporal records of individual animals at unprecedented resolution and allowing a considerable increase in knowledge of the movements, habitat use, and migratory behavior of many species (Hart & Hyrenbach, 2009; Kays et al., 2015; Scarpignato et al., 2023). With these advances, tracking data are making a growing contribution to conservation research and policy (Fraser et al., 2018; Hays et al., 2019; Lahoz‐Monfort & Magrath, 2021). For instance, they are used to identify sites of importance for biodiversity (Davies et al., 2021a) and improve coordination of international efforts for migratory species (Guilherme et al., 2023).

Shorebirds, also known as waders (order Charadriiformes), are a widespread group of birds that depend mainly on wetland and grassland habitats (Livezey, 2010; Sutherland et al., 2012). Many shorebirds are migratory, and some perform among the most impressive migrations in the animal kingdom (Alves et al., 2016; Battley et al., 2012; Conklin et al., 2017). Despite covering vast distances during their migrations, many species depend on a restricted set of feeding and resting sites to which they may be highly faithful throughout their lives (Dias et al., 2006; Gill et al., 2019), meaning local changes can have wide‐ranging repercussions on shorebird populations (Burton et al., 2006; Nightingale et al., 2023). Over the past few decades, human activities have had a significant impact on shorebird habitats worldwide (Davidson, 2014; Santos et al., 2022); nearly half of the species are in decline (IUCN, 2024). Tracking data have improved understanding of shorebird ecology and conservation, uncovering patterns of habitat use (e.g., Linhart et al., 2022; Schwemmer et al., 2016) and migratory routes and timing (e.g., Carneiro et al., 2019; Chan et al., 2019; Zhu et al., 2021) and informing protected area design (Choi et al., 2019).

Although high‐quality evidence is essential for implementing effective conservation strategies (Sutherland et al., 2004), much scientific data collected have quickly been lost or are difficult to access because of a lack of future‐focused data‐archiving practices (Whitlock, 2011). Registering, archiving, and sharing of biologging data via online platforms have been proposed to help address conservation evidence gaps (Carneiro et al., 2024; Davidson et al., 2025; Rutz, 2022; Sequeira et al., 2021). In line with this recommendation, tracking data used in scientific publications are increasingly being stored in online repositories, including generalist archives, such as Zenodo (https://zenodo.org/) and Dryad (https://datadryad.org/), and specialized platforms for data from animal‐borne sensors, such as Movebank (https://movebank.org) (Kays et al., 2022). A recent review showed that 52% of migratory shorebird tracking datasets from North America (including unpublished data) are open access (Scarpignato et al., 2023); however, the proportion of shorebird‐tracking data that are archived in online repositories globally and their characteristics remain unknown.

Tracking wild animals provides many benefits to ecology and conservation, but it does not come without its costs. The upfront costs of funding a tracking project are often prohibitive, particularly in the context of conservation, where there is a trade‐off in allocating limited resources between research (e.g., purchasing and deploying tracking devices) and management (Buxton et al., 2020). The potential impact of tracking on individuals is another important issue to consider, especially when studying small populations (Bodey et al., 2018; Fiedler, 2009; Geen et al., 2019). Therefore, to reduce impacts and unnecessary redundancy in tracking effort and to prioritize resources, it is crucial for researchers and managers to have an overview of what data currently exist. There has been no global overview of shorebird‐tracking studies. Such an overview is the first step to understanding existing disparities and achieving more globally coherent efforts to fill knowledge gaps, as has been done for seabirds (Bernard et al., 2021).

We reviewed the scientific literature and data from Movebank studies to evaluate patterns emerging from global efforts to track shorebird species. First, we considered interspecific variation in the number of published tracking studies (hereafter, *publications*) to identify how knowledge disparities may vary with shorebird characteristics, such as taxonomic family, body size, and conservation status. We then explored geographic variation in the number of tracking publications to identify regions where knowledge may be concentrated or lacking. We also assessed the extent to which data used in publications were reported as archived in online repositories. Finally, we sought to identify a set of priority species for potential future tracking research based jointly on their conservation status and population trend, quantity and spatial coverage of existing tracking data, and potential usefulness of tracking for improving their conservation status.

## METHODS

### Literature search

We reviewed the peer‐reviewed literature following the method used by Bernard et al. (2021). Our objective was to find and retrieve information from all publications reporting results from the use of miniaturized electronic devices (i.e., very‐high‐frequency radio transmitters [VHF], PTTs, GPSs, and light‐level geolocators [GLSs], hereafter *tracking devices*) to track the movements of shorebirds. We searched 2 databases: Thomson Reuters’ Web of Science (WOS) (http://apps.webofknowledge.com/) and Scopus (https://www.scopus.com). We considered 195 extant species of shorebirds in the families Burhinidae, Charadriidae, Dromadidae, Haemotopodidae, Ibidorhynchidae, Pluvianellidae, Pluvianidae, Recurvirostridae, Rostratulidae, and Scolopacidae, as listed by Birdlife International and Handbook of the Birds of the World (2022). We did not review the 33 species in the families Chionidae, Pedionomidae, Thinocoridae, Jacanidae, Turnicidae, and Glareolidae.

We searched for the English common name and scientific name of each species in the title, abstract, and keywords and for at least one of the following terms in the full text: *argos*, *biologging*, geolocat*, *GPS*, *GLS*, *PTT*, *satellite*, *telemetry*, track*, or *VHF*. The search string was (“*common name*” OR “*Latin name*”) AND (*GLS* OR *GPS* OR *PTT* OR *VHF* OR *ARGOS* OR *biologging* OR track* OR geolocat* OR *satellite* OR *telemetry*).

Using the above‐listed search terms, a list of publications was identified for each species for both databases (WOS and Scopus). The full text of each publication was inspected by either G.M. or J.N. We retained only those publications in which results from tracking devices to study shorebird movements were reported. The data were collected up to July 2023; only articles published before this date were included.

### Review of tracking publications

For each publication containing shorebird tracking data, we recorded the following information: species and taxonomic family studied, years of device deployment, country of deployment, number of individuals from which tracking data were obtained (or if the entire dataset was from previously published data, the number of individuals analyzed), device types used (i.e., VHF, PTT, GPS, GLS), whether the data presented were original (entirely or in part) or previously collected, and whether data were reported as archived in an online database (if so, which database). Publications reporting use of only previously published data were included in some analyses because relevant aspects of the data may have been only partially reported in each study (e.g., local movements reported in the first study and migration routes in a subsequent study). We also recorded the main migratory flyway used by the studied species based on the flyway classification of BirdLife International (2010). We designated the study flyway location based on explicit indication of the flyway used by the tracked birds. In cases lacking this information, flyways were determined by examining the overall itinerary of bird movements (determined from geographic coordinates or places indicated in the publication). If some points appeared outside the identified flyway but only marginally or occasionally, these deviations were considered negligible. For studies covering more than one flyway, we registered each flyway used. For all publications that contained the positions of tracked birds, we visually identified the broad latitudinal range covered by the data by assigning the study to 30° latitudinal bands in each flyway.

To explore how species’ traits and conservation status may influence tracking effort, we used the classification of migratory behavior of each species (i.e., nonmigratory, elevational, or full migrant [IUCN, 2024b]), its global conservation status (critically endangered [CR], endangered [EN], vulnerable [VU], data deficient [DD], near threatened [NT], or least concern [LC]), and its population trend (decreasing, stable, increasing, or unknown) according to the International Union for Conservation of Nature (IUCN) Red List (IUCN, 2024). We further classified each species by average body mass (Marchant et al., 2010) into the 4 size categories used by Scarpignato et al. (2016): small, <100 g; medium, 100–300 g; large, 300–500 g; and very large, >500 g.

### Search of the Movebank database

Because not all tracking data on shorebirds may appear in the peer‐reviewed literature, we complemented this information by querying the largest database for tracking data, Movebank, to identify additional tracking studies on shorebirds. Per species, we recorded the number of Movebank studies and, if permissions were set appropriately, the number of individuals tracked. After doing so, we found that the information we extracted from the literature (i.e., the number of publications and individuals tracked per species and family) correlated strongly with the patterns we found on Movebank, suggesting that the peer‐reviewed literature provides a representative, although not comprehensive, picture of shorebird tracking efforts generally (details in Appendix S1). Therefore, we used the information from Movebank to identify tracked shorebird species not appearing in the literature and to supplement our estimates of the intensity of tracking performed on them (i.e., the number of studies).

### Conservation‐priority species for tracking

To identify species that could be prioritized for future tracking efforts, we ranked species based on their conservation status, quantity of existing data, coverage of the flyways in which they are present, tracking publications (see “Geographic and taxonomic coverage”), and the potential usefulness of tracking for improving their conservation status, as indicated by the latest IUCN Red List assessments for each species (IUCN, 2024). To identify an initial list of priority species for further consideration, we first identified all species currently listed as globally threatened (i.e., CR, EN, VU) or DD or assessed as NT with a declining population trend. Next, we identified species meeting either of the following criteria: ≤2 original‐data publications or datasets on Movebank (referred to as *studies*) or ≤50% of the flyways in which they are present with any tracking publications based on visual assessments of histograms of each metric (Appendix S2).

For all species meeting the initial selection criteria, we examined the “research needed” and “conservation actions needed” sections of their IUCN Red List assessment to identify stated knowledge gaps that could be informed by tracking. We considered any stated need related to understanding migration, habitat use, or the identification of important sites for conservation as relevant knowledge gaps. For example, a need to “identify key sites” would qualify, but a need to “protect important sites” that have already been identified across the range would not. Any species with limited tracking data or geographic coverage with at least one research or conservation action need addressable by tracking was then included in the final list of species of highest priority.

### Geographic and taxonomic coverage

To assess the coverage of shorebird diversity across the world by tracking, we first estimated the number of shorebird species migrating through (or resident in) each of the major flyways. For migratory species, we used the lists of species on BirdLife International's Data Zone flyway pages (e.g., https://datazone.birdlife.org/flyway/factsheet/east‐atlantic), which represent species regularly migrating through each flyway. We then assigned resident species to flyways when ≥10% of their global range overlapped the flyway area, based on species range maps (Birdlife International & Handbook of the Birds of the World, 2022). Five threatened species (Chatham Islands oystercatcher [*Haematopus chathamensis*], Chatham Islands snipe [*Coenocorypha pusilla*], shore plover [*Thinornis novaeseelandiae*], Tuamotu sandpiper [*Prosobonia parvirostris*], and Saint Helena plover [*Charadrius sanctaehelenae*]) were excluded from this flyway coverage analysis because they are island residents that do not occur in the 8 BirdLife flyways.

To evaluate the taxonomic comprehensiveness of tracking in each flyway, we calculated the proportion of species present in each flyway that feature in at least one publication. We then calculated the total number of fully migratory, threatened, and declining species per flyway and the proportion appearing in tracking publications. To identify variation in tracking coverage within flyways, we subdivided each flyway into 30° latitudinal bands and identified species from the full flyway list with ≥1% of their global distribution in each band. Then, using just the publications with a spatial representation of the tracking data (i.e., a map), we calculated the proportion of species tracked in each flyway band. We made the above calculations based on original data publications and, to avoid duplication, included only those publications in which previously published data were used for which the full spatial extent of the tracking data was not shown in the original publications.

We carried out all analyses with R 4.5.0 (R Core Team, 2025) and mapped spatial data with QGIS 3.44.3 (QGIS Development Team, 2025).

## RESULTS

### Development of shorebird tracking

**FIGURE 1:**
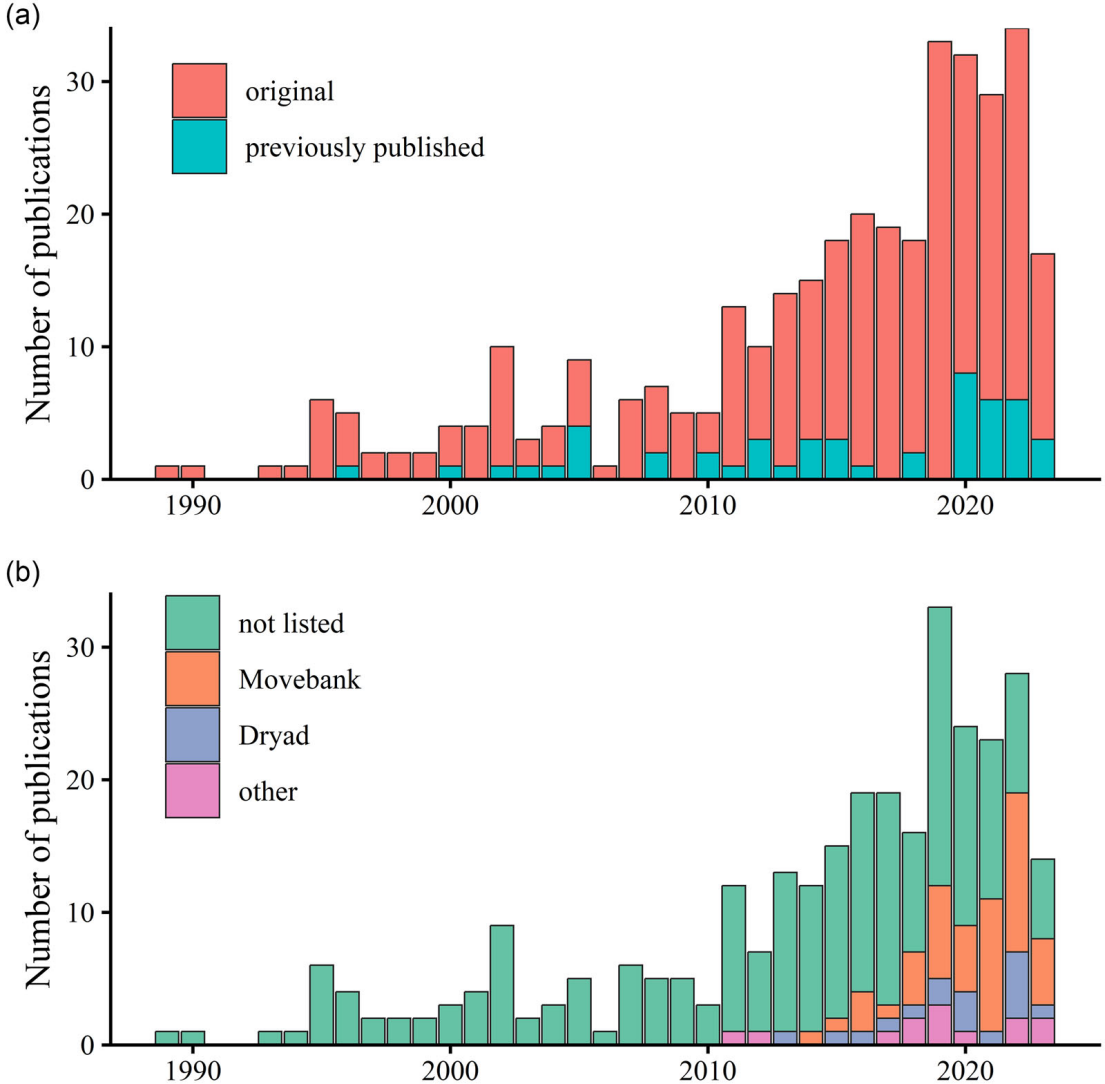
Number of shorebird tracking publications published per year from 1989 to 2023 by (a) whether they included original (entirely or in part) or previously published data (*n* = 351) and (b) online repository where, for original‐data publications (*n* = 301), archiving the analyzed data was reported.

We reviewed 351 scientific publications on the tracking of shorebirds published from 1989 to July 2023 (Figure 1; Appendix S3). We recorded 301 publications reporting novel tracking data (86%). The remaining 50 represented analyses performed on datasets that had appeared previously in publications in their entirety (Figure 1a). The annual publication of studies reporting tracking data has increased over the last 30 years. There was only one publication from 1989, and 34 were published in 2022.

Among the publications, tracking data from shorebirds were first reported as being archived in repositories in 2011 (Figure 1b). From 2015, data archiving was increasingly reported. Twenty‐one of the 28 (75%) original‐data publications from 2022 had archived data. Overall, 79 of the 301 (22.5%) original‐data publications we identified reported the data as archived (15.9% on Movebank and 6.5% on Dryad) (Figure 1b). This percentage increased to 29.7% when we considered only the 259 original‐data articles published since 2011.

### Variation among species, families, and device types

**FIGURE 2:**
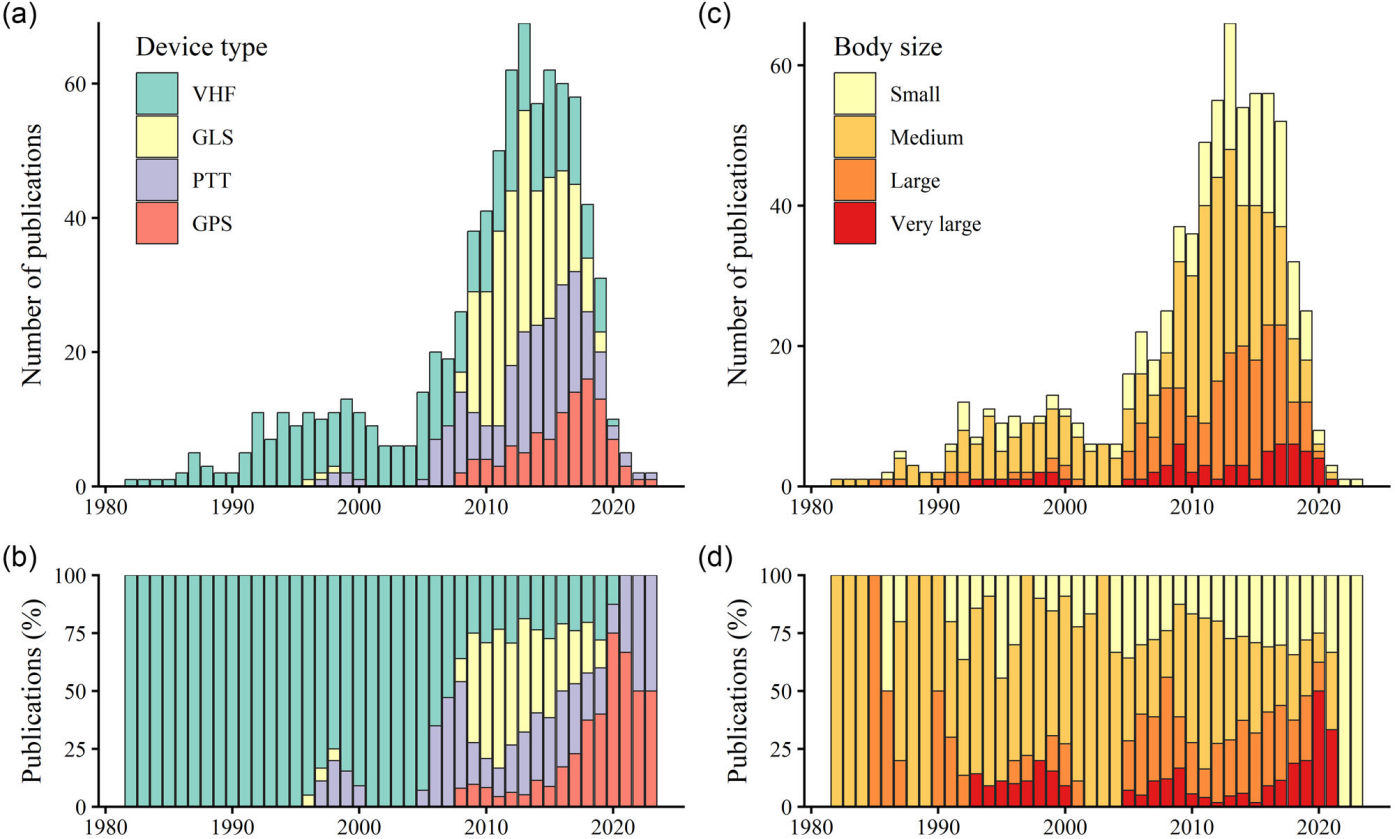
For publications reporting deployment of tracking devices on shorebirds over time, (a) total number of publications reporting the type of tracking devices deployed, (b) size category of the species studied (small, <100 g; medium, 100–300 g; large, 300–500 g; very large, >500 g), and (c) relative percentage of publications reporting deployments on species of different body size categories by device (VHF, very‐high‐frequency radio transmitters; GLS, light‐level geolocators; PTT, platform transmitter terminals; GPS, global positioning systems). The years correspond to those when transmitters were deployed on individuals, rather than the year of publication. Publications are only those that reported at least some original data (i.e., that had yet to appear in the literature up to that point) and that reported the year of tracking device deployment (*n* = 299).

Publications reported tracking 73 different shorebird species, representing 37.4% of the 195 shorebird species we focused on here. We further identified 40 tracking studies of 25 additional shorebird species on Movebank—meaning that about half of these shorebird species (49.7%) had likely yet to be tracked (Appendix S1). Of the 73 tracked species appearing in publications, most were the focus of only 1–5 publications (i.e., including original data and previously published data publications [Figure 2a]). Ten species featured in >10 publications, and 4 were the focus of >20 publications (red knot [*Calidris canutus*], black‐tailed godwit [*Limosa limosa*], dunlin [*Calidris alpina*], and whimbrel [*Numenius phaeopus*]).

Scolopacidae species were the most studied on average, with over 4 times the number of publications as the second‐most studied family, Charadriidae (Table 1; Figure 2a), although there were large variations among species within families. Families with few species were not present in any publications, although we found one study on Movebank for Dromadidae, Rostratulidae, and Pluvianellidae (Table 1). More than 93% of the species appearing in publications were fully migratory. These represented 61% of the 195 shorebird species we reviewed (Table 1). Nonmigratory species represented only 7% of the tracked species (5 of 73 species). No publications focused on the 8 elevational migrant species.

**TABLE 1:** Tracking effort on shorebirds per family based on a review of the scientific literature (articles published before July 2023) and Movebank data.

Family	Number of species	Fully migratory species (%)	Species in tracking publications (%)	Species in Movebank database (%)	Number of publications	Mean publications per species (SD), maximum number
Scolopacidae	91	82	48	54	301	3.3 (6.1), 32
Charadriidae	71	55	30	23	73	1.0 (2.2), 12
Burhinidae	10	20	10	20	7	0.7 (2.2), 7
Haemotopodidae	9	33	33	22	15	1.7 (3.9), 12
Recurvirostridae	7	57	57	43	11	1.6 (1.8), 5
Other^a^	7	57	0	43	0	–

^a^ Families with fewer than 5 species: Dromadidae, Ibidorhynchidae, Pluvianellidae, Pluvianidae, and Rostratulidae.

The main type of tracking device deployed in the early period of shorebird tracking (1982–2009) was VHF radio transmitters, which are still in use (Figure 2a). From circa 2006, the number of studies reporting deployments increased rapidly, particularly using GLS and PTT devices. The use of GPS transmitters started increasing in 2016, and after 2018, they became the primary tracking device used (Figure 2a).

Most tracking publications were on medium‐sized shorebirds (*n* = 123). After 2005, the size of species tracked became more varied. An increasing number of publications focused on smaller‐ and larger‐bodied species (Figure 2b). We found that 85% of publications indicated GLS devices were deployed to study small‐ and medium‐sized species. The PTT and GPS devices were predominantly used on large and very‐large species (67% and 80% of publications, respectively), although both began being deployed on small‐bodied species after 2012 and 2018, respectively (Figure 2c).

### Spatial patterns in tracking effort

**FIGURE 3:**
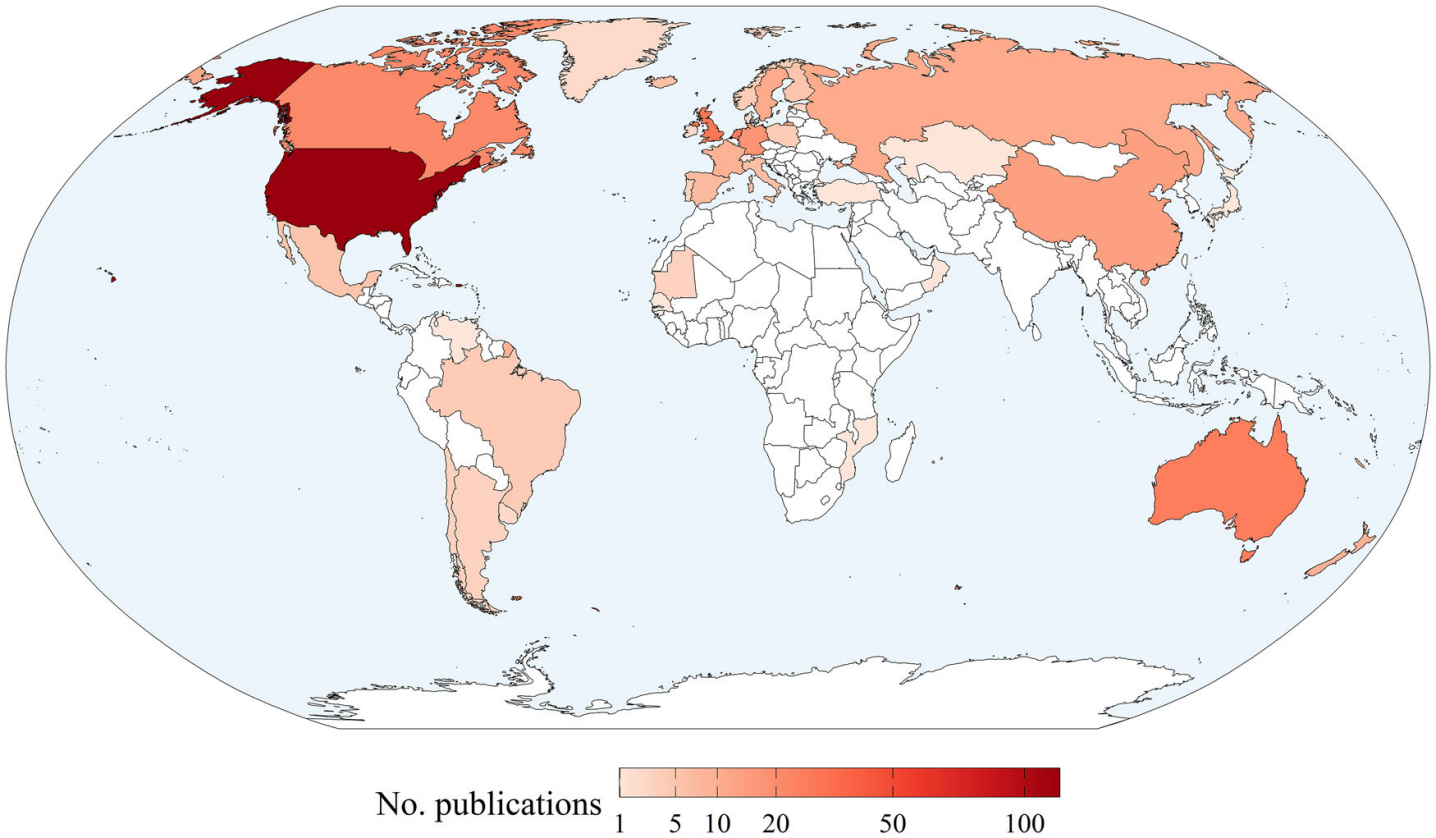
Geographical variation in the number of peer‐reviewed publications reporting tracking device deployments on shorebirds since 1989 (*n* = 39 countries with deployments) (scale, square root; white, countries lacking deployments appearing in tracking publications). The publications included are only those that reported using at least some original data (i.e., that had yet to appear in the literature up to that point, *n* = 301).

The data used in tracking publications were derived from shorebirds tagged in 39 countries (Figure 3), with the majority in North America, Europe, and Australia (88.4%). The country with the most publications reporting original data use was the United States (116 publications), followed by the United Kingdom (28 publications) and Australia (26 publications). Elsewhere, 14 publications reported data from birds tagged in China, and 13 publications contained information from birds tagged in 5 South American countries (Argentina, Brazil, Chile, Uruguay, and Venezuela). Deployments in 4 African countries (Guinea‐Bissau, Mauritania, Mozambique, and Senegal) resulted in 5 scientific publications.

**FIGURE 4:**
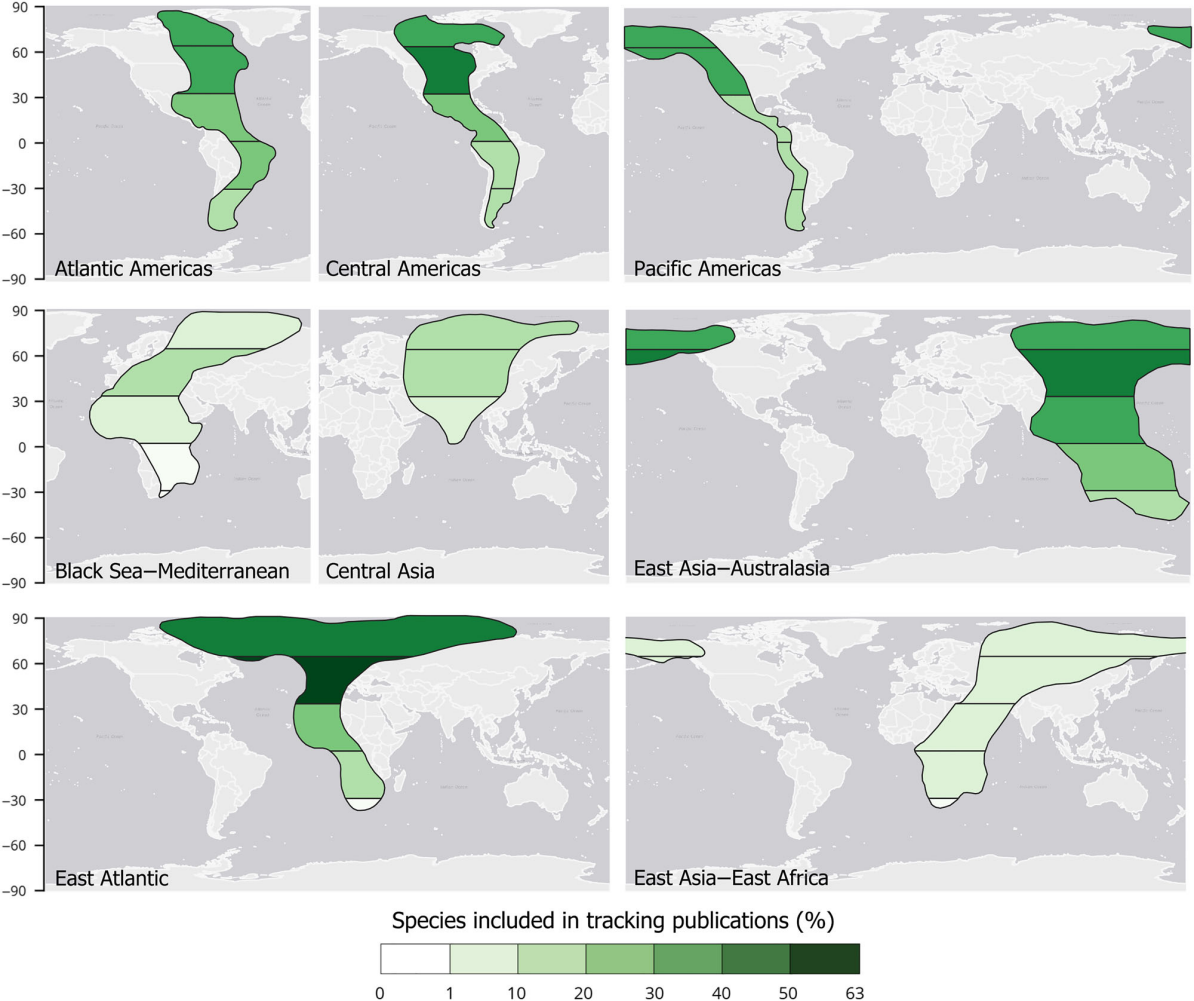
The percentage of species present in 30° latitudinal bands of each flyway (white‐to‐green polygons) that appear in tracking publications with maps showing the locations of tracked shorebirds (*n* = 190). The publications used to infer the existence of data in flyway bands included studies in which original data were analyzed and those that used previously published data.

The proportion of species tracked differed among the 8 flyways (Table 2). The Central Asia, Black Sea–Mediterranean, and East Asia–East Africa flyways had the lowest proportions of tracked species, whereas the East Atlantic, Pacific Americas, and Atlantic Americas flyways had the highest (Table 2). Tracking effort also varied latitudinally. Northern temperate regions (30°–60° N band) had higher proportions of species studied than tropical and polar latitudes in most flyways, a pattern that was particularly apparent in the East Atlantic flyway (Figure 4; Appendix S4).

**TABLE 2:** Variation among factors examined in a review of shorebird tracking research by flyway.

Flyway	Number of publications^a^	Species with publications, total species in flyway (% in flyway)^b^	Threatened species with publications, total in flyway (% in flyway)^b^	Declining species with publications, total in flyway (% in flyway)^b^	Migratory species with publications, total in flyway (% in flyway)^b^
East Atlantic	126	27, 57 (47)	1, 2 (50)	19, 25 (76)	27, 41 (66)
Atlantic Americas	71	22, 54 (41)	2, 9 (22)	16, 35 (46)	22, 46 (48)
Pacific Americas	47	25, 62 (40)	3, 7 (43)	17, 36 (49)	24, 53 (45)
East Asia–Australasia	71	29, 96 (30)	6, 18 (33)	21, 51 (41)	25, 61 (41)
Central Americas	34	15, 53 (28)	4, 10 (40)	12, 31 (39)	15, 43 (35)
Central Asia	4	5, 50 (10)	0, 7 (0)	2, 29 (7)	5, 45 (11)
Black Sea–Mediterranean	7	4, 57 (7)	0, 5 (0)	3, 25 (12)	4, 38 (11)
East Asia–East Africa	6	5, 72 (7)	1, 8 (12)	5, 35 (14)	5, 51 (10)

^a^ Includes publications based on original data and publications in which data came from other published sources or databases where the flyway was used by the birds tracked in the publication.

^b^ Total species in flyway is those regularly present in the flyway. Presence based on data and species range maps from BirdLife International and Handbook of the Birds of the World (2022).

### Priority shorebird species for future tracking

Of the 36 shorebird species classified as threatened, 13 featured in tracking publications and an additional 4 species appeared on Movebank. Across these 17 tracked species, 15 were fully migratory. Publications on threatened species took place in the East Asia–Australasia flyway, in the 3 flyways in the Americas, in the East Asia–East Africa flyway (Table 2), and on 2 species distributed across multiple migratory flyways. Of the 97 species with declining populations, 49 (50.5%) were included in at least one publication, and an additional 17 species had data on Movebank (Appendix S1). The East Atlantic and Pacific Americas flyways had the highest proportions of declining species with tracking publications (Table 2).

We identified 48 species that met the initial criteria for conservation prioritization, of which 16 species may benefit the most from future tracking (9 Scolopacidae, 5 Charadriidae, 1 Rostratulidae, and 1 Pluvianellidae) (Table 3; Appendix S2). Among the highest priority species, the tracking‐relevant research needs most frequently reported in the IUCN assessments related to life history and ecology (8 species) and species distribution (7 species). Four of these species had conservation actions needed related to identifying sites and 2 to identifying habitats. Of the 16 species of highest priority for future tracking, 8 were fully migratory, and 1 was an elevational migrant (diademed plover [*Phegornis mitchelli*]). Nine species had at least part of their range in the East Asia–Australasia flyway. Distributions of these species were highly concentrated in Southeast Asia, Indonesia, and Australia. Four species were endemic to South America (imperial snipe [*Gallinago imperialis*], diademed plover, Fuegian snipe [*Gallinago stricklandii*], and Magellanic plover [*Pluvianellus socialis*]), and one was endemic to Madagascar (black‐banded plover [*Charadrius thoracicus*]).

**TABLE 3:** Highest priority species for future tracking studies identified based on qualitative and quantitative criteria.

Common name	Scientific name	Trend	IUCN Red List status	Body size^a^	Migration	Number of publications	Movebank studies	Flyways with publications (%)^b^	Research needed^c^	Conservation actions needed^c^
Curlew sandpiper	*Calidris ferruginea*	Decreasing	VU	Small	Full	1	6	20	1.2 Distribution: Conduct research to better understand the species’ dependence on key migratory staging sites	
Buff‐breasted sandpiper	*Calidris subruficollis*	Decreasing	VU	Small	Full	3	0	50	1.2 Distribution: Document migration routes; determine important breeding, wintering, and staging areas of the species; document movement of birds in each life cycle stage	1.2 Habitat: Conserve key breeding, staging and wintering sites
Malaysian plover	*Charadrius peronii*	Decreasing	NT	Small	No	0	0	0	1.2 Distribution: Conduct surveys breeding and wintering grounds to estimate size of population and its specific habitat preferences	
Black‐banded plover	*Charadrius thoracicus*	Decreasing	VU	Small	Full	0	0	0	1.2 Life history and ecology: Collect data on species’ behavioral ecology, including mating system and competition	
Latham's snipe	*Gallinago hardwickii*	Decreasing	NT	Medium	Full	0	0	0	1.3. Life history and ecology: Determine movement ecology and identify core habitats in Australia	1.1 Site: Identify and protect nationally important sites in Australia and important staging sites
Imperial snipe	*Gallinago imperialis*	Decreasing	NT	Medium	No	0	0	0	1.2 Distribution: Search for species in habitat 1.3 Life history and ecology: Research species’ life history	
Wood snipe	*Gallinago nemoricola*	Decreasing	VU	Medium	Full	0	0	0	1.2 Life history and ecology: Research species’ ecological and habitat requirements, particularly tolerance of habitat degradation in wintering areas	
Fuegian snipe	*Gallinago stricklandii*	Decreasing	NT	Medium	Full	0	1	0		1.1 Sites: Protect areas of important habitat
Asian dowitcher	*Limnodromus semipalmatus*	Decreasing	NT	Medium	Full	0	1	0	1.2 Distribution: Conduct surveys to improve knowledge of breeding and wintering grounds	
Diademed plover	*Phegornis mitchellii*	Decreasing	NT	Small	Elevational	0	1	0		1.2 Habitats: Effectively protect significant areas of habitat at key sites in both strictly protected areas and community‐managed multiple‐use areas
Magellanic plover	*Pluvianellus socialis*	Stable	VU	Small	Full	0	1	0	1.3 Life history and ecology: Study species’ ecology	1.1 Site: Increase protection at key breeding and wintering sites
Australian painted‐snipe	*Rostratula australis*	Decreasing	EN	Medium	No	0	0	0	1.3 Life history and ecology: Locate regularly used habitat in northern Australia and determine how and why these wetlands are used; identify wetlands for management in drier years and drought refuges; undertake research to determine movements and improve knowledge of habitat preferences.	1.1 Sites: Protect and manage principal breeding and wintering sites and, as a precautionary measure, identify and protect additional habitat used by the species in the last 10 years
Moluccan woodcock	*Scolopax rochussenii*	Decreasing	VU	Medium	No	0	0	0	1.3 Life history and ecology: Establish basic demographic parameters for the species	
Javan woodcock	*Scolopax saturata*	Decreasing	NT	Medium	No	0	0	0	1.2 Distribution: Conduct surveys to estimate the size of the population and the extent of its distribution	
Hooded plover	*Thinornis cucullatus*	Decreasing	VU	Medium	No	2	0	100	1.3 Life history and ecology: Study demographic trends, including population size, sex ratio, breeding success, growth rate, and location of key breeding lakes and winter flocking sites; investigate breeding success in Western Australia	
Sociable lapwing	*Vanellus gregarius*	Decreasing	CR	Medium	Full	1	1	50	1.2 Distribution: Continue research in Kazakhstan (and initiate in Russia) on breeding habits, habitat requirements, and migration, including color ringing and satellite tracking to determine movements	

Abbreviations: CR, critically endangered; DD, data deficient; EN, endangered; IUCN, International Union for Conservation of Nature; NT, near threatened; VU, vulnerable.

^a^ Definitions: small, <100 g; medium, 100–300 g; large, 300–500 g; very large, >500 g.

^b^ Percentage of flyways in which a species is present and for which there are tracking publications.

^c^ Text drawn directly from IUCN species account—indicates listed research or conservation needs with a potential to be addressed using electronic tracking. Numeric codes refer to the levels in the IUCN conservation actions (version 2.0) and research needed (version 2.0) classification schemes (https://www.iucnredlist.org/resources/classification‐schemes).

## DISCUSSION

Tracking studies on shorebirds increased in frequency, as has been reported for other groups of birds (e.g., Iverson et al., 2023) and for biologging more generally (Kays et al., 2015). Although the reporting of data archiving in shorebird tracking publications has increased since this practice began, we estimated that just one‐third of articles have reported doing so since then.

A small number of species dominated the shorebird tracking literature; many species are yet to be tracked. We also detected a clear pattern of more publications from birds tagged in North America, Europe, and Australia, indicating geographic bias in the general understanding of shorebird movements. However, our literature search was limited to academic articles published in English, meaning we did not consider datasets appearing in publications written in other languages (Amano et al. 2021), in the gray literature, or in studies not yet published. We supplemented our literature review by identifying additional datasets on Movebank, the database we found to be most commonly used to archive shorebird tracking data (Figure 1), thereby providing a more accurate picture of tracking effort globally (Appendix S1).

Early shorebird tracking research relied on VHF devices, which could be used on all sizes of shorebirds but provided only local movement information (Warnock & Takekawa, 2003). Expansion of the Motus Wildlife Tracking System, a system for automatic detection of radio telemetry devices, now allows the study of movements at larger geographic scales, which may in part explain the continued importance of VHF technology (Taylor et al., 2017). Since the early 2000s, we have found an overall diversification in both the types of devices being deployed and the body sizes of birds being tracked. Among the publications we reviewed, GLS devices were deployed on small‐ and medium‐sized species, and GPS and PTT devices were used on the largest species until recently (Gould et al., 2024; Scarpignato et al., 2016). These patterns likely reflect both choices made by researchers to avoid using excessively large transmitters that may alter behavior or reduce survival of their study subjects and the continuing development of ever‐smaller devices (Bodey et al., 2018; Geen et al., 2019).

Four species in the family Scolopacidae were particularly well studied; some were included in several dozen publications. The red knot, dunlin, and whimbrel all occur in several flyways across the regions where most tags are deployed, likely resulting from efforts focused on multiple populations of the same species. However, almost all publications on the black‐tailed godwit referred to the nominate subspecies *Limosa limosa limosa*, and tracking efforts largely focused on Western Europe. The other 3 subspecies (*L. l. islandica*, *L. l. bohaii*, and *L. l. melanuroides*) were represented by just 2 publications (Nightingale et al., 2024; Zhu et al., 2021). Thus, the existence of many tracking studies of one population does not necessarily translate into comprehensive knowledge of a species, similar to when studies are biased toward one sex or one life stage (often adult individuals) (Benett et al., 2019). Nevertheless, the large quantity of knowledge acquired by repeatedly tracking these species, which were generally among the earliest to be studied with tracking, has helped in the development of field and analytical methods that have likely facilitated subsequent research (e.g., Beal et al., 2025; Gregory et al., 2023).

Most of the tracking publications we reviewed targeted migratory species. This may reflect the inherent difficulties of studying long‐distance migration with traditional methods (e.g., mark–recapture) and may have led to avian migration researchers being among the earliest adopters of tracking technology (Ropert‐Coudert & Wilson, 2005). This pattern might also be explained by the fact that sedentary shorebirds tend to occur in the tropics, where we found that little deployment of tags has occurred.

Tracking publications focused disproportionally on declining species; 49.7% of the world's shorebird populations are in decline (IUCN, 2024). We found that 67% of tracked species have declining populations. This may be because conservation motivates research in this field. Several publications were explicitly conservation oriented. Researchers used tracking data to address the challenges of conservation (e.g., Exo et al., 2016) and to promote conservation programs and strategies (e.g., Huysman et al., 2022; Navedo & Ruiz, 2020).

### Geographic scope of shorebird tracking

We identified a greater amount of shorebird tracking data from the Northern Hemisphere and a bias toward North America, Europe, and Australia in the deployment of devices (Figure 3), as has been found for ecological data more generally (Hughes et al., 2021). These results are in line with other reviews, which, for example, show that 93% of transmitter deployments on small‐bodied birds take place in the Northern Hemisphere (Iverson et al., 2023), where most bird ringing also occurs (Bairlein, 2003). This disparity may partly be explained by the fact that in 62% of the publications we reviewed, tracked shorebirds were tagged on the breeding grounds, which for long‐distance migrants are often at high latitudes in the Northern Hemisphere (Iverson et al., 2023; Kraaijeveld, 2014).

Economic inequalities can also help explain geographic biases (Amano & Sutherland, 2013). The high cost of tracking equipment, often exceeding USD1000 per device (Gould et al., 2024), makes them cost prohibitive for many researchers worldwide, particularly in countries with limited financial resources and investments in environmental research. The East Asia–Australasia flyway was the most latitudinally equitable flyway in tracking publications. At least 20% of occurring species were tracked in each latitudinal band, likely because most deployments occurred at nonbreeding grounds and passage areas in the lower latitudes (Figure 4). As a general pattern, the Black Sea–Mediterranean, Central Asia, and East Asia–East Africa flyways were understudied in terms of the number of publications and species studied.

It may be beneficial for the shorebird research community to consider approaches to redress the global imbalance in access to tagging technologies, which results in a lack of data for many species with conservation needs. These problems are not unique to tracking research, which is embedded in wider political and economic structures that maintain global inequities (e.g., Trisos et al., 2021). Nevertheless, we recommend that researchers and funders consider how resources, capacity, and access might be expanded to include studies focusing on species occurring in less wealthy countries. For instance, studies of migratory populations should aim to include practitioners and knowledge derived from across the species’ distribution. For this to become a reality, further support is needed for researchers in the less‐studied latitudinal ranges of the flyways, especially regarding the financing of tag acquisition. Funding bodies and journals should also consider promoting studies of understudied species and regions.

### Priority species for future tracking

By considering the extinction risk status, reported conservation‐related knowledge gaps, and state of current tracking for each shorebird species, we derived a relatively short list of species that could be considered priorities for future tracking research. The priority species identified were generally medium sized, making them likely suitable for current tracking technologies. In most cases, other species in the same genus as our priority species are already the subject of numerous publications, suggesting opportunities for knowledge sharing in designing pilot programs.

Although many of the species meeting our initial inclusion criteria were endemic to islands (Appendix S2), few were assessed as having research or conservation needs related to tracking because often their major sites and threats are known (in particular, introduced mammals and habitat loss) (IUCN, 2024). In these cases, conservation budgets may be more appropriately directed to effective site management (Buxton et al., 2020). By contrast, our prioritization for tracking highlighted pinpointed species that undertake long‐distance migrations and species with lesser‐known life histories, such as the Asian dowitcher (*Limnodromus semipalmatus*). Most occurred in the less‐studied regions, especially the East Asia–Australasia flyway or intertropics, reflecting the general geographic pattern in ecological knowledge gaps (Hughes et al., 2021) (Figures 3 & 4). The nonmigratory priority species we identified were mostly range restricted, occurring in regions or countries where there is little capture and tracking of shorebirds, such as in Southeast Asia and South America. Furthermore, species with cryptic behavior (i.e., nocturnal species or species that inhabit densely vegetated areas) and species that occur in low densities were overrepresented in our list of priority species, such as snipes *Gallinago* spp., woodcocks *Scolopax* spp., and painted snipe *Rostratula* spp., which reflects the difficulty of studying such species (Lindsey, 2009; Rasmussen et al., 1996).

Our list of priority species may be regarded as conservative, as we did not consider the need to expand protection of sites and habitats as indicating a need for tracking. However, although it may be possible to identify priority sites based on existing data (e.g., from counts [Rodkey et al., 2024]), the effectiveness of protection depends on including a comprehensive range of sites and habitats used throughout the annual cycle (Choi et al., 2019) and considering movements between them (Beal et al., 2025; Nightingale et al., 2023). Therefore, it is likely that tracking data could be a useful complement to existing information in many more cases, especially where it is difficult or dangerous to conduct direct surveys, as long as its collection does not delay or divert resources from implementing necessary management actions (Buxton et al., 2020). Nevertheless, other species may also be considered priorities in particular regions or as new knowledge becomes available—for example, species that are declining rapidly may also benefit from study before they are classified as threatened.

### Data management in shorebird tracking

Capturing and fitting wildlife with tracking devices may have negative effects on the studied individuals (Bodey et al., 2018); therefore, maximizing the use or reuse of data (and thus minimizing the number of individuals captured and tagged) should be considered an ethical obligation (Arrondo & Pérez‐García, 2025; Eren & Beaulieu, 2023). Making data available for reuse is therefore a powerful way for researchers and others who deploy tags to maximize the conservation benefits of their work. Even for those who do not make data available in online repositories, there exist regional initiatives that collate published and, in many cases, unpublished tracking data to inform on‐the‐ground conservation. For instance, in North America, the Shorebird Science and Conservation Collective works to reuse shorebird tracking data and translate it into conservation‐relevant outputs (Harrison et al., 2024).

Our review showed that a minority of studies currently report archiving data. Although such data may be archived after publication, this complicates tracing their contribution to knowledge gain over time. Data archiving is time‐consuming, requires the development of specific skills, and offers rewards to data owners that may not be immediately obvious (Michener, 2015; Whitlock, 2011). Existing initiatives to increase archiving are generally top‐down, such as open data mandates from journals or funding bodies. As a result, tracking data are sometimes stored in generalist repositories, such as Zenodo or Dryad, which can make it challenging to find and access the data, highlighting the need for a dedicated database or register of shorebird tracking data. Bottom‐up initiatives originating within tracking communities can play a valuable role in creating useful databases (Carneiro et al., 2024) and promoting cultural change (Aubin et al., 2020). Such initiatives often adhere to the principles of findability, accessibility, interoperability, and reusability (FAIR) by offering standardized data formats and promoting best practices for data description, transfer, usage, and archiving (Davidson et al., 2025). To these ends, the International Wader Study Group has recently begun the Global Wader Tracking Data Project (https://www.globalwader.org), an initiative to promote the registration and archiving of shorebird tracking data collected by academics, conservationists, and volunteers (Nightingale, 2023).

Shorebird tracking data represent an enormous and growing resource for science and conservation. They provide evidence for both the conservation of these species and the ecosystems of which they are a part. Given the increasing impact and potential of reusing tracking data stored in online repositories to inform conservation, we emphasize the need to improve coordination among teams of shorebird researchers to deploy tags strategically and to archive tracking data used in peer‐reviewed studies so their utility can be maximized in the future. By reviewing both the peer‐reviewed literature on shorebird tracking globally and the largest database of animal tracking data, we identified existing taxonomic and geographic disparities and highlighted species that may especially benefit from future tracking research. These results may help guide the development of a coherent global strategy for tracking this group of species amid the ongoing global changes in land use and climate and accelerating biodiversity loss.

## Supporting information




**Supporting Information**: cobi70211‐sup‐0001‐AppendixS1.docx

**Supporting Information**: cobi70211‐sup‐0002‐AppendixS2.docx

**Supporting Information**: cobi70211‐sup‐0003‐AppendixS3.xlsx

**Supporting Information**: cobi70211‐sup‐0004‐AppendixS4.docx
