# Supplementary material for: Global review of shorebird tracking data to identify research gaps and conservation priorities
Source: Conserv Biol. 2026 Jan 14;40(1):e70211. doi: 10.1111/cobi.70211 (PMC12856813; doi:10.1111/cobi.70211)
Supplement: Supplementary file 1 — Supporting Information: cobi70211‐sup‐0001‐AppendixS1.docx [file COBI-40-e70211-s003.docx]

# Appendix S1: Comparison of shorebird tracking data extracted from the literature with Movebank

As not all tracking data on shorebirds may appear in the peer-reviewed literature, we complemented the information gathered from the literature with a query of the largest database for tracking data, Movebank, to identify additional tracking studies on shorebirds.

We searched the Movebank API on 28^th^ May 2025 using the API query (login credentials needed to access):

https://www.movebank.org/movebank/service/direct-read?entity_type=study

which returned all findable studies in the repository (n=7720) among which we subsequently identified all studies containing at least one shorebird genus amongst the taxa listed for the study in question (n=497). Among these studies, there were a total of 78 shorebird species covered. We calculated the number of studies per species by totalling all studies listing that species. To determine the number of open access studies per species (i.e., studies for which the tracking data are downloadable), we totalled the number of studies with Creative Commons licenses.

To estimate the number of tracked individuals per species on Movebank, we summed up the total number of individuals reported in each study's metadata. When a study includes deployments on several different species, however, it is not possible to identify the specific number of tracked individuals per species, unless the data are available for download under Creative Commons licenses or is shared directly with the user’s Movebank account. For open access multi-species studies, we downloaded the deployment information using the function *movebank_download_deployment()* from the *move2* R package (Kranstauber et al. 2024). We identified 64 multi-species studies, of which just 6 (9.4%) were open access, restricting the comprehensiveness of our calculations of the number of individuals tracked per species.

To characterize the degree of similarity in the composition of datasets appearing in the literature with datasets present on Movebank, we first tested the correlation between the number of species tracked per family identified in our literature review with the number of species tracked per family present in the Movebank database. Next, we tested the relationship between the number of publications and Movebank studies per species, as well as the number of individuals tracked per species across publications with the number of Movebank deployments. We tested correlations using Spearman’s ranks.

**Results**

We found 40 Movebank studies relating to 25 species for which no publications were found at the time of our literature review (July 2023; Table A1). Vice versa, 20 species appear in peer-reviewed publications that do not have studies on Movebank, as of May 2025. On Movebank, we found studies relating to 7 of the 16 priority species (44 %) identified for future work: Fuegian Snipe *Gallinago stricklandii*, Asian Dowitcher *Limnodromus semipalmatus*, Hooded Plover *Thinornis cucullatus*, Sociable Lapwing *Vanellus gregarious,* Curlew Sandpiper *Calidris ferruginea,* Diademed Plover *Phegornis mitchellii* and Magellanic Plover *Pluvianellus socialis*.

The per-species number of publications was significantly correlated with the number of Movebank studies (Spearman’s *r* = 0.47; *S* = 122435; *p* < 0.001) as was the number of individuals in tracking publications with the number of Movebank deployments (*r* = 0.49; *S* = 13870; *p* < 0.001) and the number of species tracked per family (R = 0.95, p < 0.001; Figure A1).


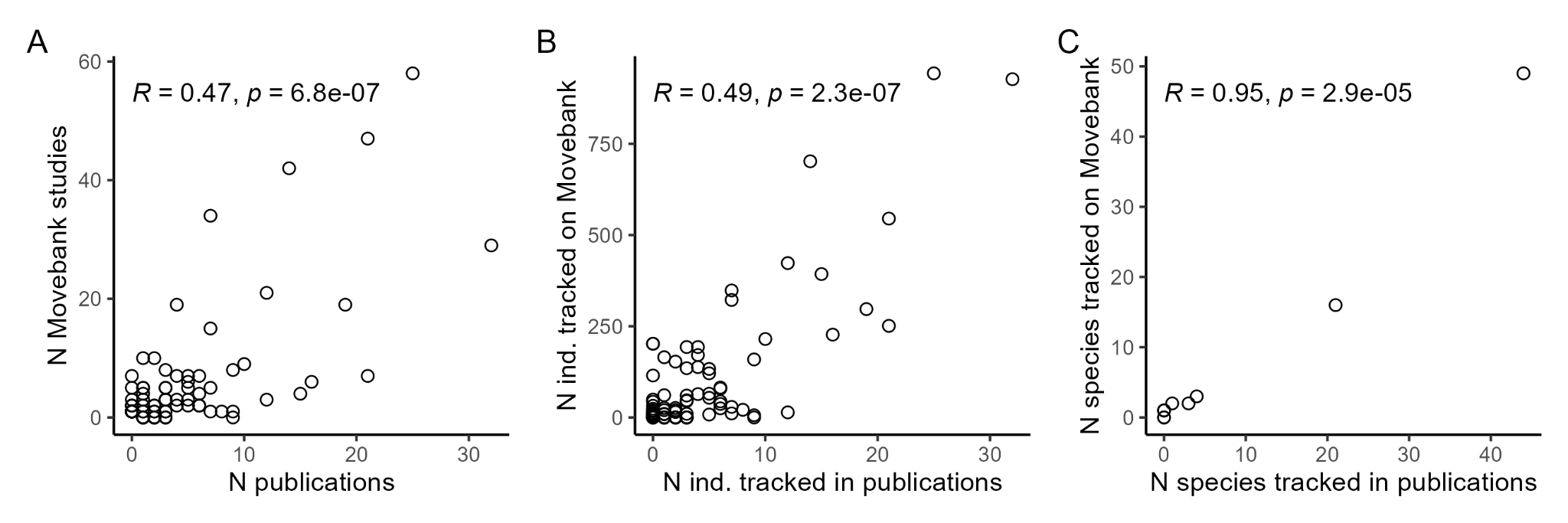


Figure A1 Variation in the (A) number of publications and Movebank studies per species, (B) the number of individuals tracked and Movebank deployments per species, and (C) the number of species tracked per family. Values in the plot refer to the Spearman’s Rank coefficient between the two variables and their significance.

**Conclusions**

The composition of tracking data for shorebirds stored on Movebank is broadly similar to the data reported in peer-reviewed literature. However, there are datasets appearing in each source of information (publications and Movebank) which are unique to each. We found that little of the data stored in Movebank is made available under open access licenses suggesting they may represent work in progress with publications forthcoming, following which data owners may make data available.

**References**

Kranstauber B, Safi K, Scharf A (2024). move2: Processing and Analysing Animal Trajectories_. R package version 0.3.0. https://CRAN.R-project.org/package=move2

Table A1. All 98 shorebird species (of the 195 shorebird species we reviewed) that feature in either the scientific literature on tracking or in a study on the online database Movebank ([www.movebank.org](http://www.movebank.org)). The number of publications and individuals tracked that were identified in the literature review (see main text) and the number Movebank studies and individuals tracked for each species are noted. Those identified as species of the highest priority for future tracking (see main text) are in **boldface**. Note that the species lacking any tracking data whatsoever will not appear in this table. NAs indicate Movebank studies for which the number of tracking deployments is not publicly available.

|  |  | Scientific literature | | Movebank | |
| --- | --- | --- | --- | --- | --- |
| Scientific name | **Common name** | **Publications** | **Individuals** | **Studies** | **Individuals** |
| *Actitis hypoleucos* | Common Sandpiper | 3 | 65 | 1 | 10 |
| *Arenaria interpres* | Ruddy Turnstone | 5 | 388 | 6 | 54 |
| *Arenaria melanocephala* | Black Turnstone | 2 | 85 | 1 | NA |
| *Bartramia longicauda* | Upland Sandpiper | 5 | 299 | 5 | 51 |
| *Burhinus grallarius* | Bush Thick-knee | 0 | 0 | 2 | 115 |
| *Burhinus oedicnemus* | Eurasian Thick-knee | 7 | 108 | 34 | 348 |
| *Calidris acuminata* | Sharp-tailed Sandpiper | 1 | 30 | 0 | 0 |
| *Calidris alba* | Sanderling | 12 | 990 | 1 | 6 |
| *Calidris alpina* | Dunlin | 22 | 1853 | 7 | 251 |
| *Calidris canutus* | Red Knot | 32 | 2532 | 29 | 927 |
| *Calidris ferruginea* | **Curlew Sandpiper** | **1** | **113** | **5** | **26** |
| *Calidris fuscicollis* | White-rumped Sandpiper | 1 | 188 | 0 | 0 |
| *Calidris himantopus* | Stilt Sandpiper | 0 | 0 | 1 | 11 |
| *Calidris maritima* | Purple Sandpiper | 2 | 110 | 0 | 0 |
| *Calidris mauri* | Western Sandpiper | 9 | 715 | 0 | 0 |
| *Calidris melanotos* | Pectoral Sandpiper | 6 | 474 | 2 | 79 |
| *Calidris minutilla* | Least Sandpiper | 1 | 62 | 0 | 0 |
| *Calidris pugnax* | Ruff | 1 | 95 | 2 | 0 |
| *Calidris pusilla* | Semipalmated Sandpiper | 15 | 2334 | 4 | 393 |
| *Calidris pygmaea* | Spoon-billed Sandpiper | 3 | 35 | 0 | 0 |
| *Calidris ruficollis* | Red-necked Stint | 2 | 126 | 2 | 20 |
| *Calidris subruficollis* | **Buff-breasted Sandpiper** | **3** | **136** | **0** | **0** |
| *Calidris temminckii* | Temminck's Stint | 2 | 60 | 0 | 0 |
| *Calidris tenuirostris* | Great Knot | 9 | 432 | 8 | 159 |
| *Charadrius alexandrinus* | Kentish Plover | 1 | 20 | 3 | 20 |
| *Charadrius bicinctus* | Double-banded Plover | 1 | 49 | 0 | 0 |
| *Charadrius dubius* | Little Ringed Plover | 3 | 133 | 0 | 0 |
| *Charadrius hiaticula* | Common Ringed Plover | 6 | 295 | 2 | 44 |
| *Charadrius leschenaultii* | Greater Sandplover | 3 | 119 | 3 | 0 |
| *Charadrius melodus* | Piping Plover | 7 | 539 | 1 | 11 |
| *Charadrius modestus* | Rufous-chested Plover | 1 | 4 | 0 | 0 |
| *Charadrius montanus* | Mountain Plover | 6 | 232 | 2 | 37 |
| *Charadrius nivosus* | Snowy Plover | 1 | 119 | 0 | 0 |
| *Charadrius obscurus* | Southern Red-breasted Plover | 0 | 0 | 1 | 1 |
| *Charadrius ruficapillus* | Red-capped Plover | 3 | 126 | 0 | 0 |
| *Charadrius semipalmatus* | Semipalmated Plover | 2 | 80 | 0 | 0 |
| *Charadrius vociferus* | Killdeer | 1 | 24 | 0 | 0 |
| *Charadrius wilsonia* | Wilson's Plover | 0 | 0 | 1 | 2 |
| *Cladorhynchus leucocephalus* | Banded Stilt | 2 | 78 | 0 | 0 |
| *Dromas ardeola* | Crab-Plover | 0 | 0 | 1 | 11 |
| *Gallinago delicata* | Wilson's Snipe | 1 | 37 | 0 | 0 |
| *Gallinago gallinago* | Common Snipe | 1 | 17 | 10 | 165 |
| *Gallinago media* | Great Snipe | 6 | 316 | 4 | 25 |
| *Gallinago megala* | Swinhoe's Snipe | 0 | 0 | 1 | NA |
| *Gallinago stenura* | Pin-tailed Snipe | 0 | 0 | 1 | NA |
| *Gallinago stricklandii* | **Fuegian Snipe** | **0** | **0** | **1** | **2** |
| *Gallinago undulata* | Giant Snipe | 0 | 0 | 1 | 24 |
| *Haematopus ater* | Blackish Oystercatcher | 3 | 39 | 1 | 26 |
| *Haematopus finschi* | South Island Oystercatcher | 0 | 0 | 2 | 202 |
| *Haematopus ostralegus* | Eurasian Oystercatcher | 16 | 401 | 20 | 419 |
| *Haematopus palliatus* | American Oystercatcher | 2 | 10 | 4 | 61 |
| *Himantopus himantopus* | Black-winged Stilt | 2 | 144 | 2 | 15 |
| *Limnodromus griseus* | Short-billed Dowitcher | 0 | 0 | 5 | 49 |
| *Limnodromus scolopaceus* | Long-billed Dowitcher | 3 | 223 | 5 | 135 |
| *Limnodromus semipalmatus* | **Asian Dowitcher** | **0** | **0** | **1** | **12** |
| *Limosa fedoa* | Marbled Godwit | 3 | 46 | 3 | 47 |
| *Limosa haemastica* | Hudsonian Godwit | 5 | 237 | 5 | 121 |
| *Limosa lapponica* | Bar-tailed Godwit | 19 | 613 | 19 | 297 |
| *Limosa limosa* | Black-tailed Godwit | 25 | 1251 | 57 | 942 |
| *Numenius americanus* | Long-billed Curlew | 4 | 58 | 7 | 193 |
| *Numenius arquata* | Eurasian Curlew | 16 | 862 | 42 | 702 |
| *Numenius madagascariensis* | Far Eastern Curlew | 8 | 144 | 1 | 21 |
| *Numenius phaeopus* | Whimbrel | 21 | 664 | 47 | 545 |
| *Numenius tahitiensis* | Bristle-thighed Curlew | 4 | 108 | 2 | 64 |
| *Phalaropus fulicarius* | Red Phalarope | 3 | 127 | 3 | 60 |
| *Phalaropus lobatus* | Red-necked Phalarope | 7 | 116 | 5 | 29 |
| *Phalaropus tricolor* | Wilson's Phalarope | 0 | 0 | 1 | 5 |
| *Phegornis mitchelli* | **Diademed Plover** | 0 | 0 | 1 | 4 |
| *Pluvialis apricaria* | Eurasian Golden Plover | 12 | 322 | 3 | 14 |
| *Pluvialis dominica* | American Golden Plover | 3 | 415 | 5 | 193 |
| *Pluvialis fulva* | Pacific Golden Plover | 6 | 154 | 6 | 62 |
| *Pluvialis squatarola* | Grey Plover | 4 | 75 | 19 | 171 |
| *Pluvianellus socialis* | **Magellanic Plover** | **0** | **0** | **1** | **NA** |
| *Recurvirostra americana* | American Avocet | 5 | 639 | 3 | 133 |
| *Recurvirostra avosetta* | Pied Avocet | 2 | 51 | 10 | 153 |
| *Rostratula benghalensis* | Greater Painted-snipe | 0 | 0 | 1 | 9 |
| *Scolopax minor* | American Woodcock | 16 | 3012 | 6 | 227 |
| *Scolopax rusticola* | Eurasian Woodcock | 11 | 787 | 9 | 215 |
| *Thinornis cucullatus* | **Hooded Plover** | **2** | **54** | **0** | **0** |
| *Tringa brevipes* | Grey-tailed Tattler | 3 | 63 | 1 | NA |
| *Tringa erythropus* | Spotted Redshank | 0 | 0 | 3 | 32 |
| *Tringa flavipes* | Lesser Yellowlegs | 4 | 324 | 3 | 138 |
| *Tringa guttifer* | Spotted Greenshank | 0 | 0 | 7 | 43 |
| *Tringa incana* | Wandering Tattler | 0 | 0 | 1 | NA |
| *Tringa melanoleuca* | Greater Yellowlegs | 0 | 0 | 1 | 19 |
| *Tringa nebularia* | Common Greenshank | 1 | 20 | 5 | 10 |
| *Tringa ochropus* | Green Sandpiper | 2 | 12 | 0 | 0 |
| *Tringa semipalmata* | Willet | 6 | 243 | 2 | 8 |
| *Tringa solitaria* | Solitary Sandpiper | 0 | 0 | 1 | 17 |
| *Tringa stagnatilis* | Marsh Sandpiper | 0 | 0 | 1 | NA |
| *Tringa totanus* | Common Redshank | 3 | 178 | 8 | 45 |
| *Vanellus cinereus* | Grey-headed Lapwing | 1 | 3 | 1 | NA |
| *Vanellus gregarius* | **Sociable Lapwing** | **1** | **29** | **1** | **9** |
| *Vanellus miles* | Masked Lapwing | 2 | 100 | 0 | 0 |
| *Vanellus spinosus* | Spur-winged Lapwing | 0 | 0 | 2 | 202 |
| *Vanellus vanellus* | Northern Lapwing | 7 | 1523 | 14 | 320 |
| *Xenus cinereus* | Terek Sandpiper | 0 | 0 | 1 | NA |
