# Supplementary material for: Global review of shorebird tracking data to identify research gaps and conservation priorities
Source: Conserv Biol. 2026 Jan 14;40(1):e70211. doi: 10.1111/cobi.70211 (PMC12856813; doi:10.1111/cobi.70211)
Supplement: Supplementary file 2 — Supporting Information: cobi70211‐sup‐0002‐AppendixS2.docx [file COBI-40-e70211-s001.docx]

# Appendix S2

A list of all species meeting the initial inclusion criteria for consideration as priority species for future tracking. We included all species that were (i) assessed as threatened (i.e., classified as CR, EN, VU) or DD; or assessed as NT with a declining population trend, with (ii) two or fewer original-data publications or datasets on Movebank (referred to as ‘studies’) OR (iii) with ≤50% of the flyways in which they are present having tracking publications.

Table A2: The most recently assessed population trend and Red List category for each species included for consideration as a potential species of highest priority for future tracking. We indicate whether conservation sites have been identified across the entire range, or only partially, and whether the species’ distribution already includes at least one Protected Area (PA). We further report the country or island group to which the species is endemic (where applicable), a classification of its migratory behaviour as assessed by the IUCN, the number of tracking publications on this species included in our review, and the percentage of the flyways in which the species is present that has tracking publications. Species we identified as the highest priorities for future tracking based on their listed research and conservation needs appear in **boldface**.

| Common name | Scientific name | Family | Trend | Status | Conservation Sites Identified | Occurs in PAs | Endemic | Migration | N Publications | N Movebank Studies | % flyways with publications | Latest date (publication or deployment) |
| --- | --- | --- | --- | --- | --- | --- | --- | --- | --- | --- | --- | --- |
| **Curlew sandpiper** | ***Calidris ferruginea*** | **Scolopacidae** | **Decreasing** | **VU** | **Entire** | **Yes** | **No** | **Full** | **1** | **5** | **20** | **2025** |
| **Buff-breasted Sandpiper** | ***Calidris subruficollis*** | **Scolopacidae** | **Decreasing** | **VU** | **Entire** | **Yes** | **No** | **Full** | **3** | **0** | **50** | **2016** |
| **Malaysian Plover** | ***Charadrius peronii*** | **Charadriidae** | **Decreasing** | **NT** | **Entire** | **Yes** | **No** | **No** | **0** | **0** | **0** | **-** |
| **Black-banded Plover** | ***Charadrius thoracicus*** | **Charadriidae** | **Decreasing** | **VU** | **Entire** | **Yes** | **Madagascar** | **No** | **0** | **0** | **0** | **-** |
| **Latham's Snipe** | ***Gallinago hardwickii*** | **Scolopacidae** | **Decreasing** | **NT** | **Partial** | **Yes** | **No** | **Full** | **0** | **0** | **0** | **-** |
| **Imperial Snipe** | ***Gallinago imperialis*** | **Scolopacidae** | **Decreasing** | **NT** | **Entire** | **Yes** | **No** | **No** | **0** | **0** | **0** | **-** |
| **Wood Snipe** | ***Gallinago nemoricola*** | **Scolopacidae** | **Decreasing** | **VU** | **Entire** | **Yes** | **No** | **Full** | **0** | **0** | **0** | **-** |
| **Fuegian Snipe** | ***Gallinago stricklandii*** | **Scolopacidae** | **Decreasing** | **NT** | **Partial** | **Yes** | **No** | **Full** | **0** | **1** | **0** | **2025** |
| **Asian Dowitcher** | ***Limnodromus semipalmatus*** | **Scolopacidae** | **Decreasing** | **NT** | **Entire** | **Yes** | **No** | **Full** | **0** | **1** | **0** | **2022** |
| **Diademed Plover** | ***Phegornis mitchellii*** | **Charadriidae** | **Decreasing** | **NT** | **Partial** | **Yes** | **No** | **Altitudinal** | **0** | **1** | **0** |  |
| **Magellanic Plover** | ***Pluvianellus socialis*** | **Pluvianellidae** | **Stable** | **VU** | **Partial** | **Yes** | **No** | **Full** | **0** | **1** | **0** | **2025** |
| **Australian Painted-snipe** | ***Rostratula australis*** | **Rostratulidae** | **Decreasing** | **EN** | **Entire** | **Yes** | **Australia** | **No** | **0** | **0** | **0** | **-** |
| **Moluccan Woodcock** | ***Scolopax rochussenii*** | **Scolopacidae** | **Decreasing** | **VU** | **Entire** | **Yes** | **Maluku Islands, Indonesia** | **No** | **0** | **0** | **0** | **-** |
| **Javan Woodcock** | ***Scolopax saturata*** | **Scolopacidae** | **Decreasing** | **NT** | **Entire** | **No** | **Indonesia** | **No** | **0** | **0** | **0** | **-** |
| **Hooded Plover** | ***Thinornis cucullatus*** | **Charadriidae** | **Decreasing** | **VU** | **Entire** | **Yes** | **Australia** | **No** | **2** | **0** | **100** | **2019** |
| **Sociable Lapwing** | ***Vanellus gregarius*** | **Charadriidae** | **Decreasing** | **CR** | **Entire** | **Yes** | **No** | **Full** | **1** | **1** | **50** | **2021** |
| Wrybill | *Anarhynchus frontalis* | Charadriidae | Decreasing | VU | Entire | No | New Zealand | Full | 0 | 0 | 0 | - |
| Peruvian Thick-knee | *Burhinus superciliaris* | Burhinidae | Decreasing | VU | Partial | Yes | No | No | 0 | 0 | 0 | - |
| Sharp-tailed Sandpiper | *Calidris acuminata* | Scolopacidae | Decreasing | VU | Entire | Yes | No | Full | 1 | 0 | 100 | 2011 |
| Broad-billed Sandpiper | *Calidris falcinellus* | Scolopacidae | Decreasing | VU | Entire | Yes | No | Full | 0 | 0 | 0 | - |
| White-rumped Sandpiper | *Calidris fuscicollis* | Scolopacidae | Decreasing | VU | Entire | Yes | No | Full | 0 | 0 | 50 | 2019 |
| Stilt Sandpiper | *Calidris himantopus* | Scolopacidae | Decreasing | NT | Entire | Yes | No | Full | 0 | 1 | 0 | 2024 |
| Least Sandpiper | *Calidris minutilla* | Scolopacidae | Decreasing | NT | Entire | Yes | No | Full | 1 | 0 | 33 | 2019 |
| Double-banded Plover | *Charadrius bicinctus* | Charadriidae | Decreasing | NT | Entire | Yes | Australia, New Zealand | Full | 1 | 0 | 100 | 2001 |
| Lesser Sandplover | *Charadrius mongolus* | Charadriidae | Decreasing | EN | Entire | Yes | No | Full | 0 | 0 | 0 | - |
| Snowy Plover | *Charadrius nivosus* | Charadriidae | Decreasing | NT | Entire | Yes | No | Full | 1 | 0 | 33 | 2021 |
| Southern, Red-breasted Plover | *Charadrius obscurus* | Charadriidae | Decreasing | CR | Partial | No | New Zealand | No | 0 | 1 | 0 | - |
| St Helena Plover | *Charadrius sanctaehelenae* | Charadriidae | Increasing | VU | Entire | Yes | St Helena | No | 0 | 0 | 0 | - |
| Killdeer | *Charadrius vociferus* | Charadriidae | Decreasing | NT | Entire | Yes | No | Full | 1 | 0 | 33 | 2002 |
| Chatham Snipe | *Coenocorypha pusilla* | Scolopacidae | Stable | VU | Partial | No | Chatham Islands, New Zealand | No | 0 | 0 |  | - |
| Beach Thick-knee | *Esacus magnirostris* | Burhinidae | Decreasing | NT | Partial | Yes | No | No | 0 | 0 | 0 | - |
| Great Thick-knee | *Esacus recurvirostris* | Burhinidae | Decreasing | NT | Entire | Yes | No | No | 0 | 0 | 0 | - |
| Madagascar Snipe | *Gallinago macrodactyla* | Scolopacidae | Decreasing | VU | Entire | Yes | Madagascar | No | 0 | 0 | 0 | - |
| Noble Snipe | *Gallinago nobilis* | Scolopacidae | Decreasing | NT | Entire | Yes | No | No | 0 | 0 | 0 | - |
| Chatham Oystercatcher | *Haematopus chathamensis* | Haematopodidae | Stable | EN | Partial | Yes | New Zealand | No | 0 | 0 | 0 | - |
| Black Stilt | *Himantopus novaezelandiae* | Scolopacidae | Increasing | CR | Partial | No | New Zealand | No | 0 | 0 | 0 | - |
| Short-billed Dowitcher | *Limnodromus griseus* | Scolopacidae | Decreasing | VU | Entire | Yes | No | Full | 0 | 5 | 0 | 2025 |
| Long-billed Dowitcher | *Limnodromus scolopaceus* | Scolopacidae | Decreasing | NT | Entire | Yes | No | Full | 3 | 5 | 33 | 2025 |
| Eskimo Curlew | *Numenius borealis* | Scolopacidae | Unknown | CR (PE) | Entire | No | No | Full | 0 | 0 | 0 | - |
| Slender-billed Curlew | *Numenius tenuirostris* | Scolopacidae | Decreasing | CR | Entire | Yes | No | Full | 0 | 0 | 0 | - |
| South American Painted snipe | *Nycticryphes semicollaris* | Rostratulidae | Decreasing | NT | Entire | Yes | No | Full | 0 | 0 | 0 | - |
| Tuamotu Sandpiper | *Prosobonia parvirostris* | Scolopacidae | Decreasing | EN | Entire | No | Tuamoto Islands, French Polynesia | No | 0 | 0 | 0 | - |
| Amami Woodcock | *Scolopax mira* | Scolopacidae | Stable | VU | Entire | Yes | Amami Islands, Japan | No | 0 | 0 | 0 | - |
| Shore Plover | *Thinornis novaeseelandiae* | Charadriidae | Increasing | EN | Partial | Yes | New Zealand | No | 0 | 0 | 0 | - |
| Spotted Greenshank | *Tringa guttifer* | Scolopacidae | Decreasing | EN | Entire | Yes | No | Full | 0 | 7 | 0 | 2025 |
| Greater Yellowlegs | *Tringa melanoleuca* | Scolopacidae | Decreasing | NT | Entire | Yes | No | Full | 0 | 1 | 0 | 2025 |
| River Lapwing | *Vanellus duvaucelii* | Charadriidae | Decreasing | NT | Entire | Yes | No | No | 0 | 0 | 0 | - |
| Javan Lapwing | *Vanellus macropterus* | Charadriidae | Decreasing | CR | Entire | No | Java | No | 0 | 0 | 0 | - |

Figure A2.1


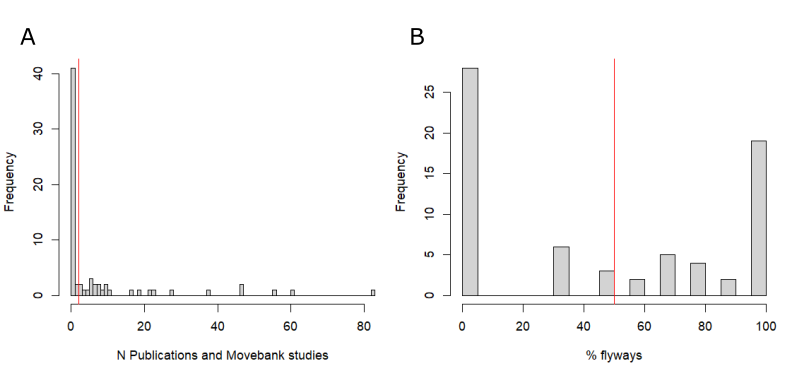


Figure A3.1. Two quantitative criteria used to identify the initial set of priority species for future tracking effort (i.e., all species listed in Table A3). (A) The total number of tracking publications and Movebank studies per species, with the chosen threshold for inclusion of ≤ 2 shown in red. (B) The percentage of flyways in which each species is present that has a tracking publication, with the chosen threshold of ≤ 50% shown in red.

Figure A2.2


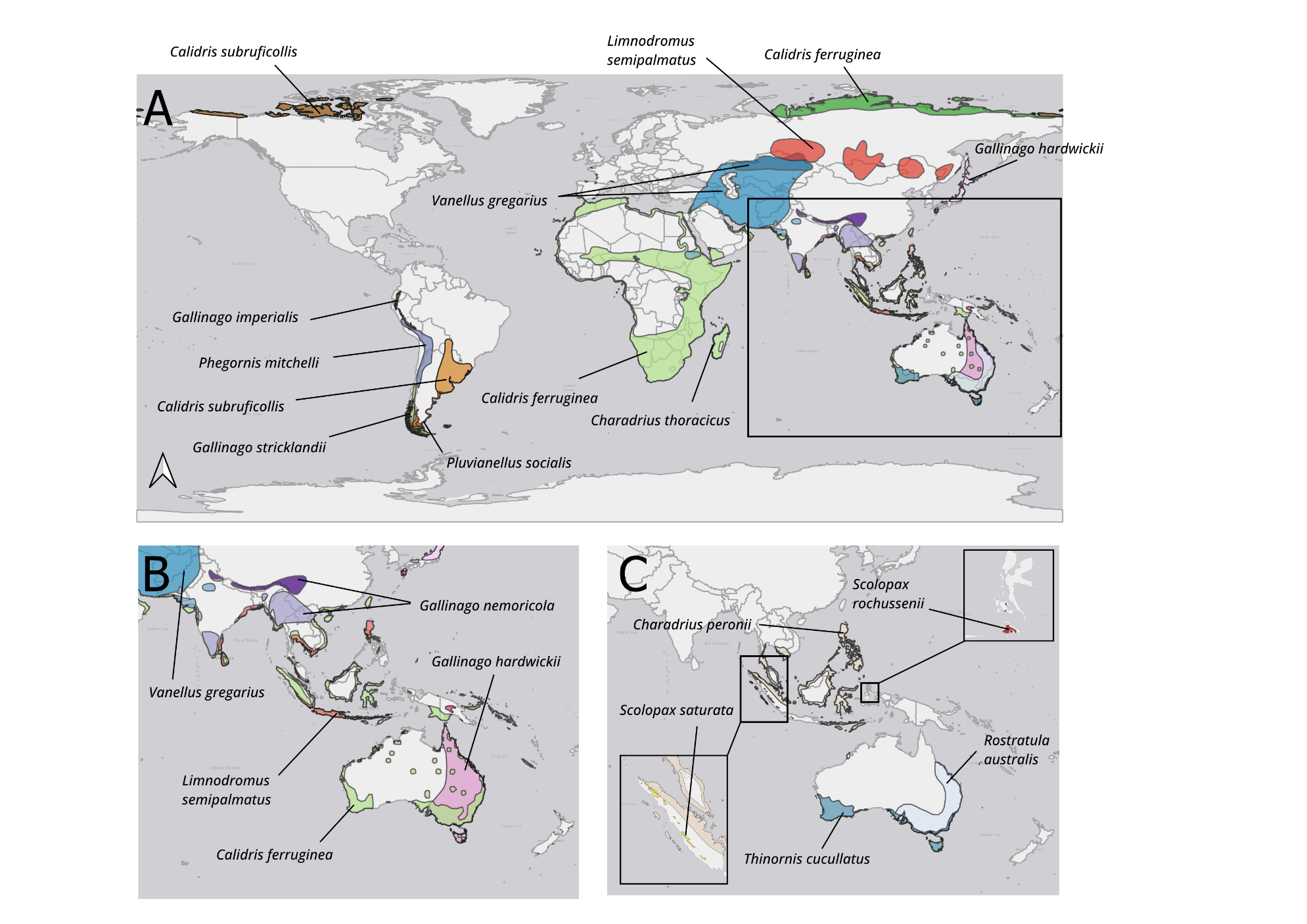


Figure A2.2: Distribution of the 16 priority shorebird species, showing (A) species with widespread distributions; and an inset highlighting migratory (B) and non-migratory (C) species in the South-East Asia and Australasia region.
