# Supplementary material for: Global review of shorebird tracking data to identify research gaps and conservation priorities
Source: Conserv Biol. 2026 Jan 14;40(1):e70211. doi: 10.1111/cobi.70211 (PMC12856813; doi:10.1111/cobi.70211)
Supplement: Supplementary file 4 — Supporting Information: cobi70211‐sup‐0004‐AppendixS4.docx [file COBI-40-e70211-s004.docx]

**Appendix S4**

Figure A4


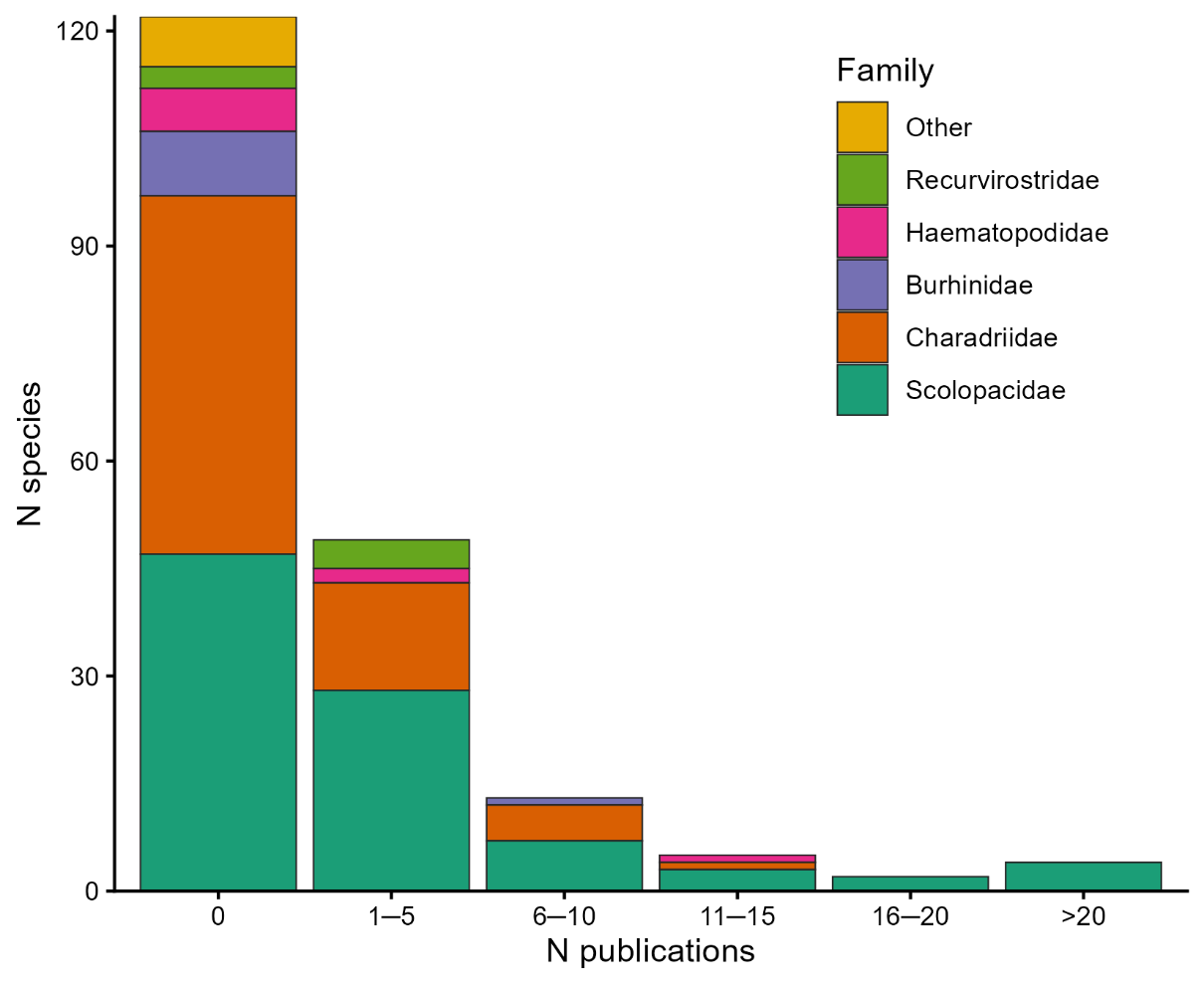


Figure A4: The number of tracking publications per shorebird species (N = 351), grouped according to taxonomic family. “Others” includes all families with fewer than five species (Dromadidae, Ibidorhynchidae, Pluvianellidae, Pluvianidae and Rostratulidae). The publications tallied here include both those reporting original and re-used tracking data.

Table A4.1: Numbers of tracked species and publications within each flyway **overall and within 30° latitudinal bands**, as calculated using all 351 publications that we identified as having analysed tracking data on shorebirds.

| **Flyway** | **Species occurring** | **Species tracked** | **-60°–30°** | **-30° –0°** | **0°–30°** | **30°– 60°** | **60°– 90°** |
| --- | --- | --- | --- | --- | --- | --- | --- |
| Atlantic America | **57** | **27** | 5 | 7 | 9 | 12 | 6 |
| Central America | **53** | **15** | 5 | 6 | 12 | 13 | 6 |
| Pacific America | **62** | **25** | 4 | 5 | 6 | 14 | 11 |
| Central Asia | **50** | **5** | 0 | 0 | 4 | 5 | 4 |
| East Asia - East Africa | **72** | **5** | 0 | 2 | 4 | 4 | 2 |
| Black Sea - Mediterranean | **57** | **4** | 0 | 0 | 4 | 4 | 1 |
| East Atlantic | **57** | **27** | 0 | 5 | 12 | 22 | 15 |
| East Asia - Australasia | **96** | **29** | 8 | 14 | 21 | 20 | 14 |

Table A4.2: Number of publications containing maps of the tracking data, which allowed for assigning the data to specific latitudinal bands of flyways used by the birds tracked (n = 190). Note that publications with data covering multiple bands will be duplicated across bands of the same flyway.

| **Flyway** | **Total Publications** | **-60°–30°** | **-30°–0°** | **0 –30°** | **30°– 60°** | **60°– 90°** |
| --- | --- | --- | --- | --- | --- | --- |
| Atlantic America | **39** | 5 | 11 | 20 | 32 | 15 |
| Central America | **42** | 5 | 6 | 12 | 13 | 6 |
| Pacific America | **40** | 7 | 7 | 8 | 23 | 14 |
| Central Asia | **13** | 0 | 0 | 4 | 5 | 4 |
| East Asia - East Africa | **12** | 0 | 3 | 4 | 4 | 2 |
| Black Sea - Mediterranean | **9** | 0 | 0 | 5 | 6 | 1 |
| East Atlantic | **54** | 0 | 6 | 29 | 70 | 29 |
| East Asia - Australasia | **77** | 13 | 28 | 43 | 44 | 30 |
|  |  |  |  |  |  |  |
